# Utility of plasma circulating tumor DNA and tumor DNA profiles in head and neck squamous cell carcinoma

**DOI:** 10.1038/s41598-022-13417-5

**Published:** 2022-06-04

**Authors:** Nobuyuki Chikuie, Yuji Urabe, Tsutomu Ueda, Takao Hamamoto, Takayuki Taruya, Takashi Kono, Kohei Yumii, Sachio Takeno

**Affiliations:** 1 Department of Otorhinolaryngology, Head and Neck Surgery, Graduate School of Biomedical and Health Sciences, Hiroshima University, 1-2-3 Kasumi, Minami-ku, Hiroshima, 734-8551 Japan; 2 Division of Regeneration and Medicine Center for Translational and Clinical Research, Hiroshima University Hospital, Hiroshima, Japan

**Keywords:** Cancer genomics, Head and neck cancer, Metastasis, Oncogenes, Tumour biomarkers, Cancer, Oncology, Cancer, Cancer genetics, Head and neck cancer, Tumour biomarkers

## Abstract

Early recurrence detection of head and neck squamous cell carcinoma (HNSCC) is important for improving prognosis. Recently, circulating tumor DNA (ctDNA) has been reported to be useful in early detection or treatment response determination in various carcinomas. This study aimed to identify the utility of ctDNA for predicting recurrent metastasis in patients with HNSCC. We collected pre-treatment tissues (malignant and normal tissues) and multiple plasma samples before and after treatment for 20 cases of HNSCC treated with radical therapy. ctDNA was detected in pre-treatment plasma in 10 cases; however, there were no significant associations with tumor recurrence and staging. During follow-up, ctDNA was detected in 5 of the 7 plasma samples of recurrent cases but not in the 13 recurrence-free cases. Moreover, there was a significant difference in post-treatment relapse-free survival time between the groups with and without detected ctDNA (20.6 ± 7.7 vs. 9.6 ± 9.1 months, respectively; log-rank test, p < 0.01). Moreover, for two of the five cases with ctDNA detected after treatment, ctDNA detection was a more sensitive predictor of recurrence than imaging studies. ctDNA detection during treatment follow-up was useful in patients with HNSCC for predicting the response to treatment and recurrent metastasis.

## Introduction

Head and neck cancer (HNC) is the seventh most common cancer worldwide, with more than 800,000 new cases diagnosed annually^1^. Head and neck squamous cell carcinoma (HNSCC) is by far the most common form of HNC. More than 60% of patients with HNSCC exhibit locally advanced or distant metastasis with a poor prognosis at the time of diagnosis. After the primary treatment of locally advanced cases, local recurrence occurs in approximately 60% of patients. Distant metastasis is also seen in up to 30% of patients with or without local recurrence. In addition, second primary cancer develops in 5–13% of the cases^2,3^.

However, although some cases of long-term survival have been reported, the prognosis for patients with recurrent metastasis remains poor. Untreated patients with recurrent or metastatic disease have a prognosis of approximately 6 months of overall survival^4^. Despite advances in disease diagnosis, treatment, and surveillance, the overall survival of patients has not significantly improved over the past 30 years. The key to improving overall and progression-free survival is the early detection of cancer and recurrent metastasis. Frequent computed tomography (CT) scans, magnetic resonance imaging, and endoscopic examinations are necessary to detect recurrence and metastasis. However, these procedures are complicated by radiation exposure and difficulty in evaluating residual disease and recurrence due to sequelae after chemoradiation therapy (CRT).

Recently, genetic testing using next-generation sequencing has become the standard for personalized medicine in various organ cancers. In HNSCC, the high frequencies of *TP53* loss-of-function mutations and *CDKN2A* inactivation are observed in smoking-related HNSCCs, and *PIK3CA* mutations, *TRAF3* deletion, and *E2F1* amplification are seen in human papillomavirus (HPV)-related HNSCCs in The Cancer Genome Atlas (TCGA) network^5^.

Moreover, recent technological advances for the detection and characterization of circulating tumor DNA (ctDNA) can enable the detection of genomic alterations through minimally invasive examinations^6^. ctDNA in plasma has been suggested as a potential biomarker for cancer detection and for monitoring disease burden and tumor response^7^. The analysis of ctDNA may be useful in adjuvant therapy, as it can identify patients at high risk for disease recurrence based on the detection of postoperative minimal residual disease (MRD). In cervical cancer, ctDNA was shown to be valuable for monitoring tumor behavior and treatment response^8^. Many patients with HNC exhibit some form of ctDNA alterations^9^. However, it remains unclear how advanced HNC can be detected using ctDNA, and investigations are required regarding the clinical significance of mutated genes. Although a few recent studies have explored ctDNA in the field of head and neck cancer, reports at the clinical level are rare, especially in Asian patients^10–13^.

In this study, we analyzed plasma cell-free DNA (cfDNA) following curative therapy for HNSCC. We aimed to identify the potential role of ctDNA in treatment monitoring and recurrence detection in patients with HNSCC and clarify the clinical utility of gene mutation analysis.

## Results

### Characteristics of patients

Twenty patients with HNSCC were included in this study. The clinical and histopathological characteristics of these patients are summarized in Table 1. The median age in our study was 65 years. Eight patients (40%) exhibited stage I HNSCC, one (5%) showed stage II, six (30%) were in stage III, and five (25%) were in stage IV. Of the 20 cases, 16 were of oropharyngeal cancer, 1 was of oral cancer, and 3 were of hypopharyngeal cancer. Of the 16 patients with oropharyngeal cancer, 11 were p16-positive.

Eleven patients were treated with a combination of cisplatin-based chemotherapy and radiation therapy, whereas nine patients were treated with radical surgery. Of the nine cases that were operated on for initial treatment, eight were negative for resection margins. One case was positive for resection margins. This case was of a woman with oropharyngeal cancer (cT4aN3bM0). The neck metastasis had invaded the internal jugular vein, and a composite resection with the internal jugular vein was performed. The patient was at high risk of recurrence and underwent postoperative adjuvant chemoradiotherapy. Regardless of the type of initial treatment, recurrence tended to be more common in patients with advanced cancers. Specifically, there was one recurrence among the nine patients in stages I and II and six recurrent or metastases among the 11 patients in stages III and IV. The median follow-up time was 24 months (range 4–44 months). The patient with the shortest follow-up time (4 months) exhibited rapidly worsening disease due to postoperative distant metastasis.

In the seven cases with recurrence, ctDNA was detected in five plasma samples during follow-up. In the non-relapsed group, ctDNA was undetectable in all 13 cases. ctDNA in pre-treatment plasma was found in 10 of 20 patients, and the detection frequency tended to increase as the disease stage progressed (see Supplementary Table S1 online).

**Table 1:** Patient characteristics.

Total cases	20
**Sex**	
Male	16
Female	4
**Age, median (range, year)**	65 (33–81)
< 65	12
65 ≦	8
**Anatomic site**	
Oropharynx	16
Oral cavity	1
Hypopharynx	13
ECOG PS0	20
**Tabaco use**	
Never	4
Current	16
**Alcohol consumption**	
Current and former use	
Yes	14
**Clinical stage at diagnosis (UICC 8th)**	
I.II	9
III.IV	11
**HPV status**	
Positive	11
Negative	5
Unknown	4
**1st line therapy**	
Operation	9
Chemoradiotherapy	11
Recurrence or metastasis	7

*UICC* Union for International Cancer Control.

### Genomic alteration (tissue somatic mutation) in patients with HNSCC

The most frequently mutated gene in the HNSCC samples was *KMT2D* (mutated in 80.0% of our cohort), followed by *PCLO* (65%), *KMT2C* (60%), *TP53* (45%), *SDHA* (45%), and *NOTCH1* (30%) (see Supplementary Figure online). When divided by HPV status, there were two of 11 cases in HPV-positive oropharyngeal cancer and six of nine cases in HPV-negative oropharyngeal cancer and oral/hypopharyngeal cancer. The percentage of *TP53* mutations in HPV-negative cases was 66%. In addition, six of all cases (30%) were positive for *NOTCH1.* The Cancer Genome Atlas Network reports that the mutation frequency of T*P53* and *NOTCH1* in HPV-negative HNSCC is 84% and 26%, respectively^5^. Mutation frequencies were similar in this study. As for *KMT2D*, Harbison et al. reported that *KMT2D* is one of the most frequently mutated genes in pharyngeal carcinoma, and we suspect that the same trend was observed in this study^14^.

### Relationship between plasma ctDNA and clinical course

ctDNA was detected in pre-treatment plasma in 10 of the 20 cases. Among the seven recurrent cases, ctDNA was detected in three pre-treatment samples. There were no significant associations with tumor recurrence and TNM stage in the detection of ctDNA at the time of initial diagnosis (Stage I–II vs. III–IV; 3/9 vs. 7/11, p = 0.36, T1–2 vs. T3–4; 5/11 vs. 5/9, p = 1.0, N0 vs. N1; 8/15 vs. 2/5, p = 1.0) Because of the different N staging between p16-positive and -negative cases, the actual number of metastatic lymph nodes before treatment was assessed using CT imaging and was not related to the detection of ctDNA. Three of the four cases with distant metastasis after treatment exhibited ctDNA in the plasma before treatment. The cases in which plasma ctDNA was detected during follow-up tended to be in the advanced stage group (III + IV vs. I + II; 5/11 vs, 0/9 p = 0.0378). ctDNA detection was also associated with local recurrence (4/6 vs, 1/14 p = 0.0139) and distant metastases (3/4 vs 1/16 p = 0.0010) (Table 2).

**Table 2:** Factors associated with ctDNA detection.

	ctDNA in pretreatment plasma	p value*	ctDNA in follow-up plasma	p value*
Gender (male vs. female)	3/4 vs. 7/16	0.582	2/4 vs. 3/16	0.248
Stage group (I, II vs. III, IV)	3/9 vs. 7/11	0.369	0/9 vs. 05/11	0.0378
T stage (1, 2 vs. 3, 4)	5/11 vs. 5/9	1.0	1/11 vs. 4/9	0.127
N stage (positive vs. negative)	8/15 vs. 2/5	1.0	4/15 vs. 1/5	1.0
Local recurrence (positive vs. negative)	2/6 vs. 8/14	0.628	4/6 vs. 1/14	0.0139
Distant metastasis (positive vs. negative)	3/4 vs. 7/16	0.580	4/4 vs. 1/16	0.001
HPV status (positive vs. negative or unknown)	5/11 vs. 6/9	0.406	0/11 vs. 5/9	0.008
Age (< 65 vs. 65 ≦)	8/12 vs. 3/8	0.361	3/12 vs. 2/8	1.0

*Fisher’s test.

Post-treatment detection of ctDNA in plasma and Fisher test were performed for each item. Post-treatment detection was associated with stage, local recurrence, and distant metastasis.

**Table 3:** Association of clinical factors with the presence or absence of ctDNA detection in follow plasma.

	Detected ctDNA in follow Plasma n = 5	Non detected ctDNA in follow plasma n = 15	p value*
Age, over 65	2/5	6/15	1.0
Stage III–IV	5/5	6/15	0.037
HPV status Positive	0/5	11/15	0.008

*Fisher’s test.

Patient characteristics.

Plasma ctDNA was detected after treatment in five of the seven recurrent cases. Four of these five cases involved distant metastases (see Supplementary Table S1 online); the remaining case was a local recurrence with extranodal extension of the cervical lymph nodes found during imaging.

**Figure 1:**
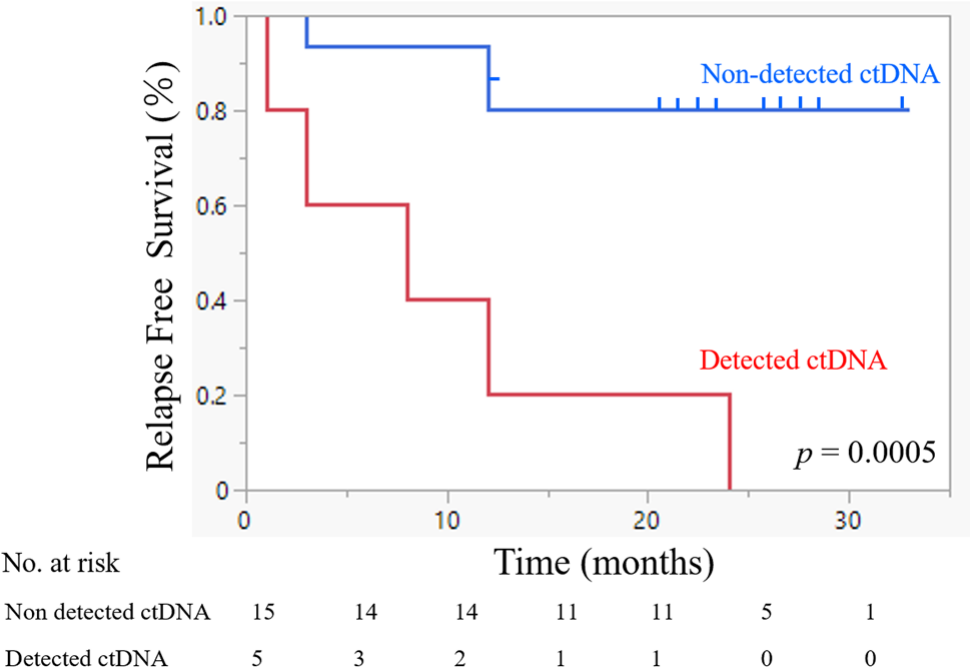
Relevance of ctDNA detection in relapse-free survival. Relapse-free survival (RFS) rate of 20 patients with head and neck squamous cell carcinoma who underwent detection of circulating tumor DNA (ctDNA) in the plasma. The time to recurrence was significantly shorter in the group in which ctDNA was detected (log-rank p = 0.0005).

**Figure 2:**
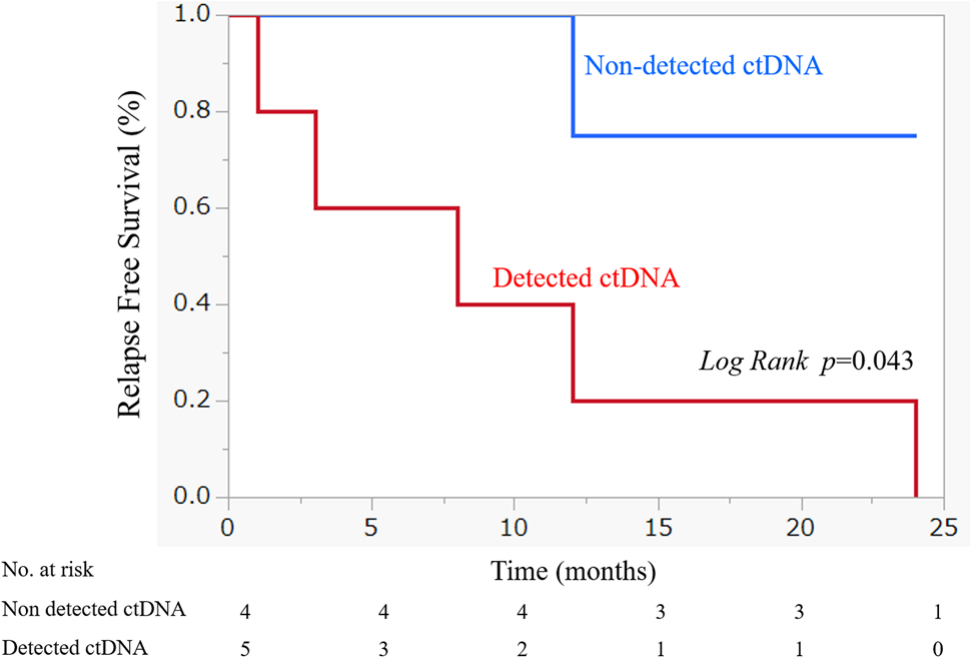
Relevance of ctDNA detection in HPV-negative patients in relapse-free survival. Relapse-free survival (RFS) rate of patients with HPV-negative head and neck squamous cell carcinoma who underwent detection of circulating tumor DNA (ctDNA) in the plasma. The time to recurrence was significantly shorter in the group in which ctDNA was detected (log-rank p = 0.043).

**Figure 3:**
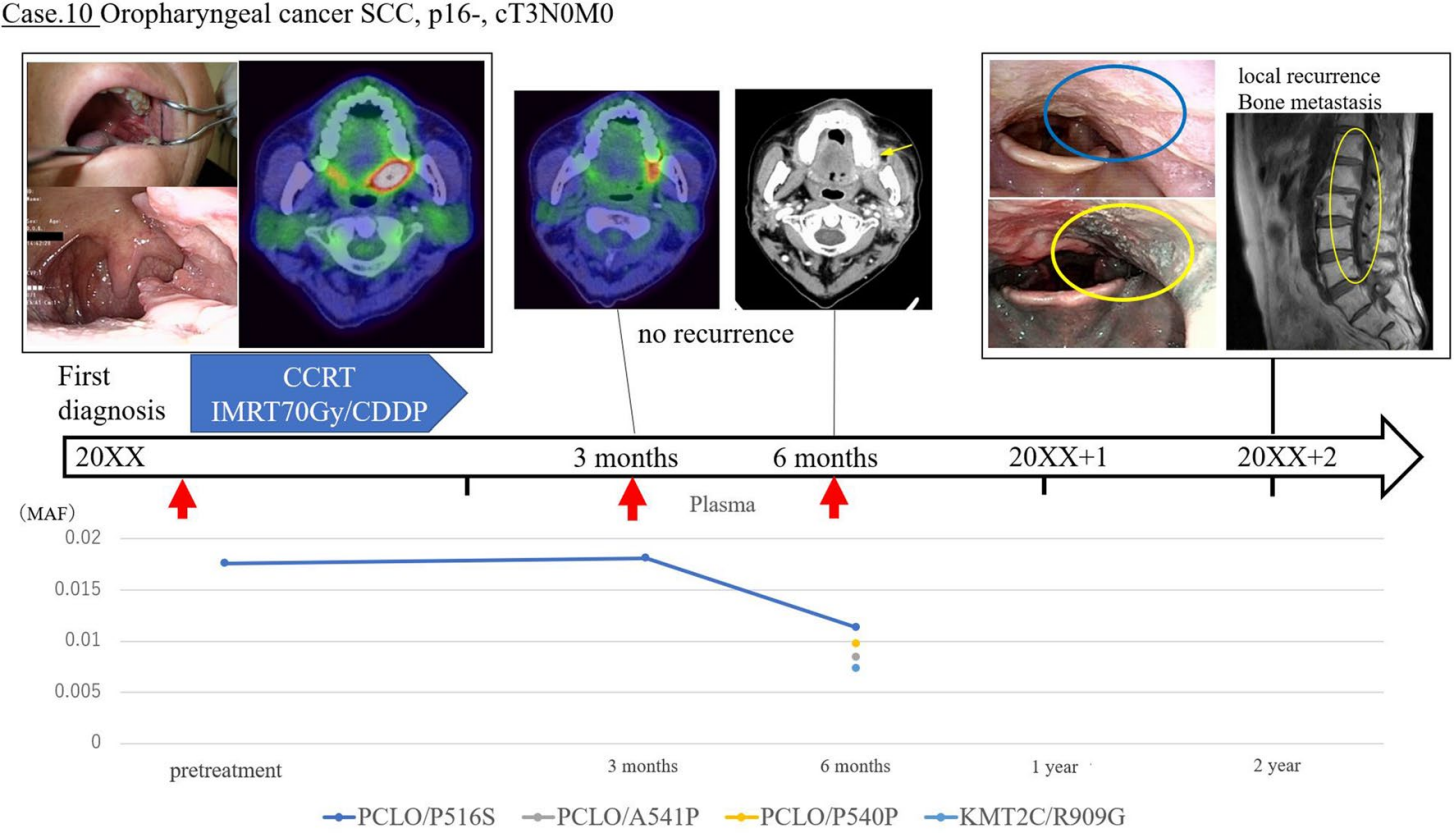
Sample case for use of plasma circulating tumor DNA to detect HNSCC (Case 10). Clinical course of patient #10. This patient exhibited a p16-negative oropharyngeal carcinoma cT3N0M0 and was treated with chemoradiotherapy. The patient was treated with a cisplatin combination. Before treatment, ctDNA was detected via *PCLO*/P516S. Post-treatment imaging studies showed that the tumor had disappeared, but ctDNA was still detected 6 months after treatment. Tumor recurrence was detected 2 years after treatment during follow-up imaging.

**Figure 4:**
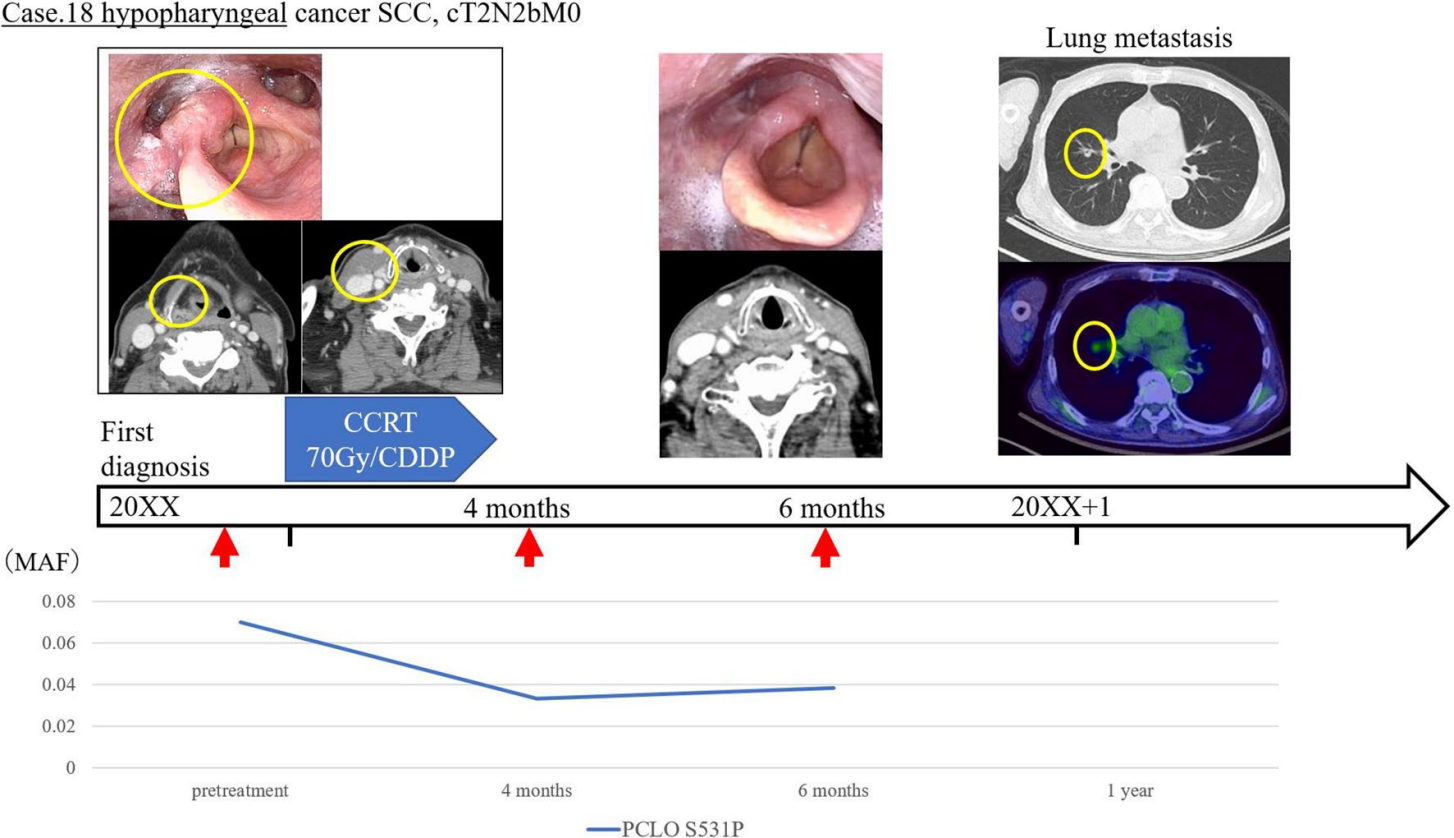
Sample case for use of plasma circulating tumor DNA to detect HNSCC (Case 18). Clinical course of patient #18. This patient exhibited a hypopharyngeal carcinoma cT2N2bM0 and was treated with chemoradiotherapy. Before treatment, ctDNA was detected via PCLO/S531P. Post-treatment imaging showed that the tumor and lymph metastasis had disappeared, but ctDNA was still detected 6 months after treatment. Lung metastasis was observed in the first year after treatment.

Conversely, of the 10 cases in which ctDNA was detected before treatment, there were seven in which ctDNA was not detectable after treatment. None of these patients experienced clinical recurrence during follow-up, and there was no significant difference in relapse-free survival (RFS) as compared to the cases in which ctDNA was not detected during follow-up. In the examination of plasma after CRT or surgery, patients with detectable ctDNA had a significantly decreased median RFS (8 months; 95% confidence interval (CI) 1–24; 60% recurrence) when compared with patients negative for ctDNA (> 33 months; 95% CI 12–NA; 13.3% recurrence; p < 0.001) (Fig. 1). Since there is a significant difference in patient background in HPV status and stage between the two groups in which ctDNA was detected in post-treatment plasma and the group in which ctDNA was not detected, we included the analysis only for the subject group with HPV-negative patient background (Table 2). The Kaplan–Meier curve of RFS in the HPV-negative group also showed a significant difference (p = 0.043 by the log-rank test) (Fig. 2). A significant difference in overall survival was not found between the cases with the presence and absence of ctDNA after treatment. Of the five recurrent cases in which ctDNA was detected after treatment, three cases were consistent with the course of existing CT imaging findings, while in two cases, the ctDNA detection was found to be more sensitive than CT or other imaging. Of these two cases with high ctDNA sensitivity, one (case 10) continued to exhibit detectable ctDNA three months after treatment, although the tumor was undetectable in CT images. This patient developed local recurrence in the pharynx and metastasis to the lumbar spine after two years. In the other case (case 18), despite the disappearance of the tumor in the CT images six months after treatment, ctDNA remained detectable, and lung metastasis was detected after 1 year (Figs. 3 and 4).

## Discussion

In this study, we investigated the clinical utility of ctDNA detection and sequencing for predicting the prognosis and/or recurrence of HNSCC. We examined cfDNA in tumor tissue samples and in pre- and post-treatment plasma samples from patients with HNSCC and found that ctDNA was more likely to be detected in more advanced cancers. In treatment-refractory cases that relapsed after curative treatment, ctDNA was detected in pre-treatment plasma. Moreover, our findings suggest that ctDNA detection after treatment is a more sensitive predictor of HNSCC recurrence and/or metastasis than CT scanning or other imaging.

ctDNA is extracellular DNA released into the bloodstream from cancer cells that have been lysed by pathological and physiological mechanisms, such as apoptosis and necrosis^15^. In contrast, cfDNA is present in non-cancerous states, derived from apoptotic and necrotic cells, and phagocytosed by macrophages and other scavenger cells under normal circumstances^16^. In cancer patients, cfDNA is composed of a combination of non-cancerous cell DNA from normal cells and ctDNA from cancer cells. Generally, ctDNA makes up 0.1–10% of total cfDNA, depending on the stage and size of the cancer and the type of cancer cells^17^. In theory, liquid biopsy can provide more complete information about total tumor volume in a patient. This is because the sample represents the DNA of all tumors theoretically present in the circulation, unlike a biopsy sample, which represents a single lesion within a single anatomical site^6^. Moreover, liquid biopsy is often more convenient than traditional biopsy and minimizes the risk to the patient. Tissue biopsy is an invasive procedure and is generally performed in a surgical setting under infiltration or general anesthesia. Due to edema after chemoradiotherapy and modified anatomy after reconstructive surgery, especially in the head and neck region, current biopsy techniques have limited reproducibility of tissue access and tumor sampling. The collection of ctDNA from plasma, however, has significant advantages in that it can be replicated spatially (given the heterogeneity of the tumor) and temporally (given the dynamic tumor changes)^18^. A simple blood draw in outpatient consultation can avoid the delays and risks associated with repeated biopsies and surgical intervention while effectively detecting tumor heterogeneity and identifying hidden metastases throughout the body^19^. Blood sampling also helps determine when to perform an effective imaging test. In this study, we showed that it was possible to detect ctDNA in the plasma of patients with HNSCC using capture-based next-generation sequencing^20–22^.

Previous studies have found patterns regarding the relationship between ctDNA and tumor stage. For example, in colorectal cancer, the detection of ctDNA has been reported to be associated with multi-organ metastasis or tumor size^23^. It is thought that this occurs because larger tumors increase the rates of apoptosis and necrosis, releasing more ctDNA than smaller tumors^24–26^. Our results also showed that ctDNA was more easily detected in advanced cancer. Hilke et al*.* reported a longitudinal analysis of ctDNA dynamics during chemoradiotherapy in 20 patients with locally advanced HNSCC treated with CRT^27^. The results showed a significant correlation between the amount of ctDNA detected and the tumor volume and revealed that the tumor allele fraction in plasma was negatively correlated with the amount of ctDNA detected. Additionally, when ctDNA was detected in the first follow-up, disease recurrence was seen. These findings support our conclusion that tumor stage is associated with detection rate in HNSCC. To date, there is little clear data on the role of ctDNA in early HNSCC, and hence, further research is required to determine the association of ctDNA with prognosis and metastasis in the early stages.

In this study, post-treatment ctDNA was detected in five (71.4%) of the seven patients with recurrent illness. In contrast, follow-up ctDNA was not detected in any case without recurrence or metastasis. The detection of ctDNA after curative treatment was significantly associated with recurrence and RFS. Our results indicate that ctDNA may be a tool to detect recurrence and could be used for the selection of adjuvant therapy. Similarly, Tie et al. reported an association between detection of plasma ctDNA and recurrence after resection of stage II colon cancer and suggested that ctDNA may help in identifying the risk of recurrence^28^. In addition, ctDNA has been reported to be a useful predictor of treatment efficacy and disease progression in cervical cancer, and for evaluation of MRD in recurrent metastasis lung cancer^8,29,30^. In HNC, Khandelwal et al. reported that the detection of ctDNA after surgery could be a prognostic prediction biomarker for cancer recurrence^31^. According to their paper, in 22 cases of oropharyngeal cancer, detection of TP53 was associated with recurrence. Because these results are similar to those of our study, we believe that ctDNA detection can be used as an indicator to predict recurrence and metastasis in patients with HNC with difficult-to-observe areas after chemoradiotherapy or surgery.

In this study, two patients with recurrent HNSCC exhibited detectable ctDNA prior to tumor detection by imaging. In these two cases, distant metastases were found by CT scans, 6 and 18 months after the detection of ctDNA at 6 months after treatment. ctDNA detection is a rapid, noninvasive, and repeatable means of measuring disease status at multiple time points and may be effective in reducing the required frequency of imaging tests, which carry the risk of radiation exposure and side effects from contrast agents. Coombes et al*.* reported that plasma ctDNA was detected prior to clinical or radiological confirmation of recurrence in 49 patients with breast cancer. In the same study, ctDNA was not detected in plasma from 31 patients without recurrence^32^. In hereditary nonpolyposis colorectal cancer, HPV16 level in cfDNA after radical radiotherapy alone or in combination with chemotherapy had been suggested to be useful as a complementary biomarker for the early identification of treatment failure^33^. These results indicate that patient-specific ctDNA analysis is a highly sensitive approach for surveillance of HNC, allowing early detection and the possibility of therapeutic intervention.

Our study has several limitations, the most crucial of which is the small number of participants due to the limited study period at a single center. Additionally, our analysis included only 71 cancer-related genes. Finally, although we were able to collect plasma samples from patients, we were not able to examine plasma over a prolonged period of time (e.g., after 1 year) due to the timing and cost of the experiment. However, our study has two major strengths. It is a rare report to examine the importance of cfDNA on a practical clinical level and the first to do so in an Asian cohort. The superiority of ctDNA detection even in a small number of cases and the fact that similar results have been obtained for other cancer types suggests that ctDNA may be superior to existing diagnostic imaging methods^34^. To address these limitations, we believe that we can expect to gain more insight into ctDNA by studying a higher number of cases, extending the observation period, and increasing the number of oncogene search items. Therefore, future studies should conduct long-term plasma ctDNA monitoring in a large group of patients. The common view of the American Society of Clinical Oncology and the College of American Pathologists is that there is still insufficient scientific evidence to use monitoring for the early detection of cancer and therapeutic efficacy^21^. Moreover, currently, there is no international standard protocol for the detection of ctDNA^35^. However, the results of this pilot study offer to improve the treatment and prognosis of HNSCC by suggesting an early detection method. Despite the current limitations regarding ctDNA analysis for early cancer detection, our findings suggest that this is a method worth developing and applying clinically.

Plasma ctDNA detection in patients with HNSCC has great potential as a noninvasive, rapid, and repeatable adjuvant tool for cancer detection and surveillance. As we rapidly approach a point of increased treatment stratification and personalization for cancer care, liquid biopsies may play a critical role in facilitating the treatment and management of HNSCC.

## Methods

### Patients and sample collection

The present study was performed at the Hiroshima University Hospital, in Hiroshima, Japan, and was approved by the Hiroshima University Human Genome Ethical Committee (Hiroshima University Hospital IRB Hi-213). Informed consent for whole-genome analysis was obtained from all patients, in accordance with the Declaration of Helsinki Ethical Principles for medical research involving human subjects. All patients presented with pathologically confirmed HNSCC, and all patients who were aged ≥ 18 years at the time of resection were recruited in this study. Patients who had a malignant tumor other than squamous cell carcinoma during histopathological examination were not included in the study. The tumor and normal tissue and blood samples of patients with performance status (ECOG PS) of 0–2 were collected before treatment. Blood was collected at regular office visits for imaging evaluation after curative therapy such as surgery or chemoradiation (3, 6, 9, or 12 months post-treatment). Tissue samples were collected from patients undergoing biopsy or surgery at the Hiroshima University Hospital from December 2017 to December 2020. A total of 10 mL of whole peripheral blood from each patient was collected into ethylenediaminetetraacetic acid tubes. However, for patient convenience and due to the timing of imaging tests, cases in which blood could not be collected on a regular basis were also included. A total of 94 distinct samples (39 tissue samples and 55 blood samples) from 20 individuals with HNSCC were obtained. Clinical data were collected from medical records and included sex, age, smoking status, alcohol consumption, p16 status, treatment history, recurrence, and TNM classification (UICC 8th)^36^. The clinicopathological characteristics of individuals included in this study are shown in Table 1.

### Sample preparation

Plasma was extracted from whole blood by centrifugation at 2302.9×*g* and 4 °C for 10 min. The plasma was aliquoted (4 mL) and frozen at − 80 °C until DNA isolation. Tissue samples were also frozen at − 80 °C until DNA isolation. The frozen plasma and tissue samples were thawed at room temperature just before DNA extraction. Plasma cfDNA plap was extracted using the Maxwell RSC Large volume cfDNA Plasma Kit (Promega, Madison, Wisconsin, USA)^37^. Tissue cfDNA was extracted using the Maxwell RSC Blood DNA Kit (Promega)^38^. Nucleic acids were automatically extracted from both samples using the Maxwell RSC Instrument. DNA concentrations were measured using a Qubit dsDNAHS Assay Kit (Thermo Fisher Scientific, Waltham, MA, USA) and Qubit 1.0 fluorometer. If necessary, the isolated DNA was concentrated by centrifugation and then stored at − 20 °C.

### Target enrichment and next-generation sequencing

DNA extracted from the tumors and control lymphocytes was fragmented into 150–200 bp segments using the SureSelect Enzymatic Fragmentation Kit (Agilent Technologies, Santa Clara, CA, USA), and libraries were constructed according to manufacturer instructions^39^. A total of 107–115 ng of DNA from all plasma samples and 115–125 ng of DNA from all tissue samples were prepared for sequencing. A total of 71 oncogenes with somatic mutation and copy number alterations of more than 5% in HNCs according to the International Cancer Genome Consortium Data Portal were identified. The exons of these oncogenes were enriched using SureSelect-XT Low Input Target Enrichment (Agilent; Table 2)^40,41^. The resulting pooled libraries underwent quality control through the High Sensitivity D1000 ScreenTape System on the 2200 TapeStation Instrument (Agilent). Sequencing was performed with paired-end reads using the NovaSeq 6000 platform (Illumina) (see Supplementary Table S2 online).

### Variant detection

Sequencing reads were aligned to the hg19/GRCh37 reference sequence and analyzed using the SureCall Software version 4.2 (Agilent)^42^. Duplicates were removed using a molecular barcode to improve mapping quality prior to variant calling. Paired-end and single analyses were used to identify single-nucleotide variants and insertions/deletions (indels) in tumors and cfDNA. Called variants were considered germline mutations if they were also present in DNA from the control lymphocytes. To reduce the false-positive rate, we set the cutoff values for somatic mutations in tumors and cfDNA as follows: read depth > 20 and forward/reverse balance between 0.25 and 0.75. We also configured the SureCall SNP caller using SureSelect default settings: variant score threshold, 0.3; minimum quality for base, 30; variant call quality threshold, 100; minimum allele frequency, 0.005; and minimum number of reads supporting variant allele, 10. Moreover, variants that (a) were repeated sequences registered in the UCSC repeat masker, (b) called as replacements, or (c) clearly identified as sequence errors in the Integrated Genomic Viewer software 2.11.4 (Broad Institute) were excluded as somatic mutation candidates in all sample types. We classified somatic mutations into three categories: (I) frameshift indels or nonsense mutations; (II) missense mutations; and (III) synonymous changes or mutations located in introns.

### Statistical analyses

Statistical analyses were performed using JMP pro version 16.0 (developed by the SAS Institute). Factors related to the detection of ctDNA were evaluated using Fisher’s exact test. RFS was evaluated using the log-rank test separately for ctDNA detection and non-detection. Results were considered statistically significant at probability values (p) < 0.05.

## Supplementary Information

Supplementary Information.

## Data Availability

The datasets that support the findings of our study are available from the authors on reasonable request. The datasets generated and/or analyzed during the current study are available in the Department of Otorhinolaryngology, Head and Neck Surgery Graduate School of Biomedical and Health Sciences Hiroshima University repository, https://jibika.hiroshima-u.ac.jp/research/documents/data%20availability%20about%20mutation%20genes%20list%20from%20utility%20of%20plasma%20circulating%20tumor%20dna%20and%20tumor%20dna%20profile%20in%20head%20and%20neck%20squamous%20cell%20carcinoma.pdf.
