# Supplementary material for: Utility of plasma circulating tumor DNA and tumor DNA profiles in head and neck squamous cell carcinoma
Source: Sci Rep. 2022 Jun 4;12:9316. doi: 10.1038/s41598-022-13417-5 (PMC9167274; doi:10.1038/s41598-022-13417-5)
Supplement: Supplementary file 1 — Supplementary Information. [file 41598_2022_13417_MOESM1_ESM.pdf]

Table S1. Patient clinical data and detected ctDNA

Case	site	gender	BI	T	N	M	Stage	HPV	Local recurrence	Distant metastasis	Plasma Before treatment	Follow Plasma
											Detection	Detection
1	Oropharyngeal	Male	1260	1	0	0	I	Negative	+	−	−	−
2	Oropharyngeal	Male	0	2	1	0	I	Positive	−	−	−	−
3	Oropharyngeal	Male	500	2	1	0	I	Positive	−	−	+	−
4	Oropharyngeal	Female	0	2	0	0	I	Positive	−	−	+	−
5	Oropharyngeal	Male	480	2	1	0	I	Positive	−	−	+	−
6	Oropharyngeal	Male	150	2	1	0	I	Positive	−	− −	−	−
7	Oropharyngeal	Male	400	2	0	0	I	Positive	−	−	−	−
8	Oropharyngeal	Male	600	2	1	0	I	Positive	−	−	−	−
9	Oropharyngeal	Male	530	2	0	0	II	Negative	−	−	−	−
10	Oropharyngeal	Male	400	3	0	0	III	Negative	+	+	+	+
11	Oropharyngeal	Male	400	4	2	0	III	Positive	−	−	−	−
12	Oropharyngeal	Female	200	4	2	0	III	Positive	−	−	+	−
13	Oropharyngeal	Male	800	4	1	0	III	Positive	−	−	+	−
14	Oropharyngeal	Male	780	4a	2	0	III	Positive	+	−	−	−
15	Oral	Female	0	3	1	0	III	Unknown	+	−	−	+
16	Oropharyngeal	Male	600	2	2b	0	IVa	Negative	−	−	+	−
17	Oropharyngeal	Female	0	4a	3b	0	IVb	Negative	+	+	+	+
18	Hypopharyngeal	Male	675	2	2b	0	IVa	Unknown	−	+	+	+
19	Hypopharyngeal	Male	345	4a	2b	0	IVa	Unknown	+	+	−	+
20	Hypopharyngeal	Male	1000	3	2b	0	IVa	Unknown	−	−	+	−

BI ＝Brinkman Index; T = tumor; N = lymph nodes; M = metastasis; Stage = Stage (UICC 8th); HPV = Human Papillomavirus

Table S2. HNSCC Targeted NGS panel

ADGRA2	AGO	ATR	BCL6	BIRC3	CASP8	CCND1	CDKN2A	CDKN2B	CREBBP
DCUN1D1	EGFR	EIF4A2	EP300	ETV5	EXT1	FAT1	FAT4	FBXW7	FGF3
FGF4	FGF12	FGF19	FGFR1	FOXL2	GMPS	GRM3	HRAS	KLHL6	KMT2C
KMT2D	LPP	LRP1B	LRRK2	MAP3K13	MECOM	MGAM	MTAC	MYC	NDRG2
NFE2L2	NOTCH1	NSD3	NUMA1	PC	PCLO	PIK3CA	PIK3CB	PRKCI	PRKDC
RAD21	RECQL4	RELN	RNF213	ROS1	RPS6KB2	SDHA	SOX2	SPEN	STAG1
TBL1XR1	TERC	TERT	TFRC	TP53	TP63	TRIP13	WWTR1	YAP1	ZNF703
1NPPL1									

The panel for this study consisted of 71 oncogene exons from the International Cancer Genome Consortium (ICGC) Data Portal that were found to have somatic mutations and copy number changes of 5% or more in head and neck cancers.

Supplementary figure: Somatic mutation in tumor tissue

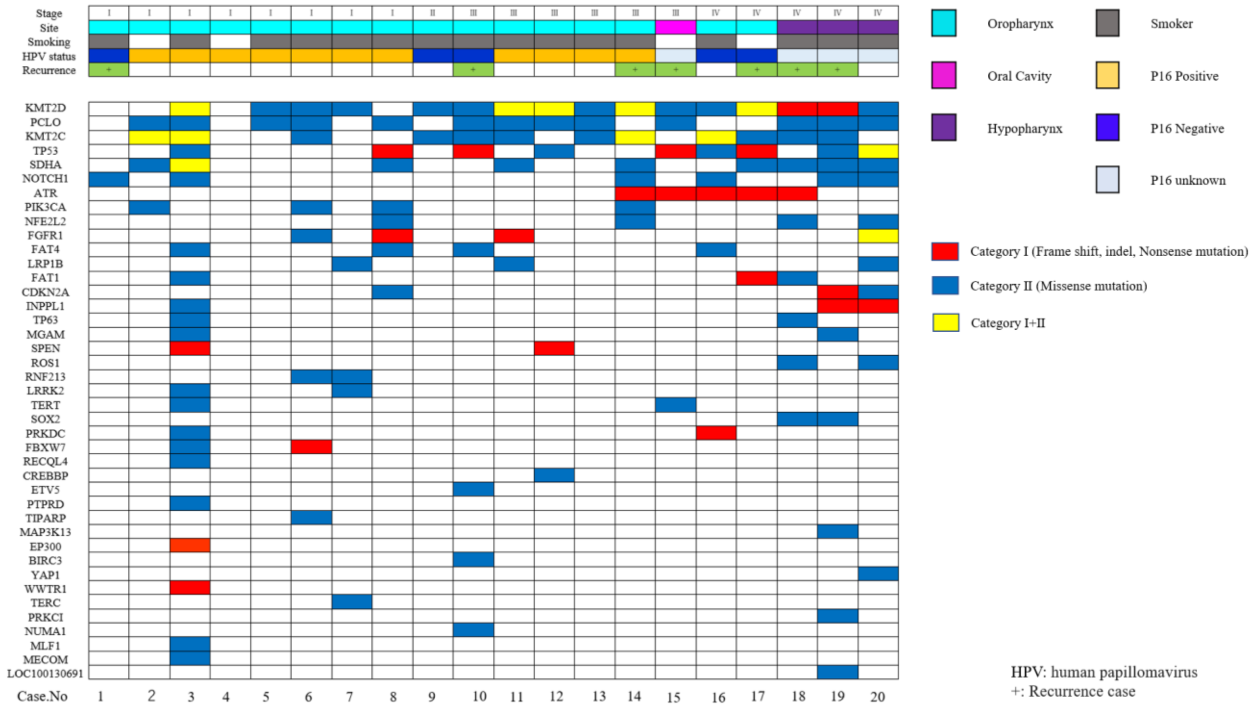

The results of mutation profiling in 20 tumor tissue samples, divided into Category I (frame shift, indel, and nonsense mutations) and Category II (missense mutation) according to the type of gene mutation. Results are shown with the clinical background of the tumor. The upper panel shows clinical backgrounds for each lesion, including tumor stage and location, human *papillomavirus* (HPV) status, and recurrence after treatment. The lower panel shows the result of the mutation pattern of each mutated gene in each tissue. Genes with somatic mutations in this study are shown on the left. Red cells, Category I mutation; blue cells, Category II mutations; yellow cells, both Category I and II mutations.
